# Cyclophilin A/CD147 signaling induces the epithelial-to-mesenchymal transition and renal fibrosis in chronic allograft dysfunction by regulating p38 MAPK signaling

**DOI:** 10.1080/0886022X.2022.2126788

**Published:** 2022-10-06

**Authors:** Xuzhong Liu, Zhiwang Tang, Xi Jiang, Tianwei Wang, Lun Zhao, Zongyuan Xu, Kun Liu

**Affiliations:** Department of Urology, Huai’an First People’s Hospital, Nanjing Medical University, Nanjing, Huai’an, China

**Keywords:** Cyclophilin A, CD147, epithelial-to-mesenchymal transition, kidney transplantation, chronic allograft dysfunction

## Abstract

**Objective:**

Our study was designed to explore the role of Cyclophilin A (CyPA)/CD147 signaling in renal allograft fibrosis and chronic allograft dysfunction (CAD).

**Materials and methods:**

A rat renal transplant model with significant CAD was successfully achieved. Renal allograft tissues and blood samples were collected. Hematoxylin and eosin, Masson’s, and immunohistochemistry staining were performed. Since CD147 is mainly expressed in the renal tubular epithelial cells, human HK-2 cells were used and intervened by specific concentrations of CyPA, and the total protein and mRNA were extracted. Western blot assay and polymerase chain reaction were performed to explore the protein and mRNA expression of CyPA, CD147, and epithelial-to-mesenchymal transition (EMT)-related biomarkers. SiRNA-CD147 and specific inhibitors of p38 MAPK were used to explore the cellular mechanisms involved in the process.

**Results:**

We have successfully established and validated a 20-week renal transplant CAD model. We observed significant distributed and expressed CyPA and CD147 in the renal allograft fibrotic tissues. We also found a significant expression of CD147 and EMT-related markers in the HK-2 cells stimulated by CyPA. The CD147 *siRNA* confirmed the previous *in vitro* results. The selective inhibition of MAPK suggested the notable role of p38 MAPK signaling pathway in the CyP/CD147 signaling involved in renal allograft fibrosis.

**Conclusions:**

Our study reported the positive relationship of CyPA-CD147 signaling with renal allograft dysfunction. The *in vitro* study suggested that CyPA-CD147 signaling induce the development of the EMT process by p38 MAPK signaling, thus contributing to renal allograft fibrosis and CAD.

## Introduction

Chronic allograft dysfunction (CAD) remains the main factor influencing the long-term allograft survival of renal transplant recipients, characterized by interstitial fibrosis (IF), tubular atrophy (TA), and glomerulitis [1,2]. While substantial studies have reported the mechanism of renal allograft fibrosis, there is still a lack of detailed and comprehensive knowledge for the CAD. It is known that several factors, such as transforming growth factor-beta 1 (TGF-β1) and tumor necrosis factor-α (TNF-α), play a crucial role in the formation of allograft fibrosis [1,3,4]. To date, the massive infiltration of myofibroblasts in renal interstitium and to a lesser extent in glomeruli was reported to be closely correlated with the development of renal fibrosis, percentage of which could proportionally indicate the severity of renal fibrosis [5]. However, the origin of myofibroblasts during the development of renal fibrosis still remained to be explored.

Epithelial-to-mesenchymal transition (EMT), characterized as the loss of epithelial cell markers (E-cadherin) and subsequently acquired the ability of mesenchymal markers [vimentin, α-smooth muscle actin (α-SMA)], has been extensively explored in the wound healing, cancer metastasis, and organ fibrosis in past decades [6]. A large number of studies have suggested the possibility of EMT in renal fibrosis *in vivo* and *vitro*, and particularly, in allograft interstitial fibrosis through classical and non-classical pathways [3,7]. The supportive evidence of EMT in renal fibrosis have been provided in a variety of basic studies using different animal models and genetic lineage tracing systems, suggesting that EMT is partially responsible for the occurrence and development of renal fibrosis [8–10]. Nevertheless, the therapeutic target and molecular mechanism involved in EMT requires further investigation.

In recent years, Cyclophilin A (CyPA), a ubiquitous intracellular protein, has been recognized to be secreted to the extracellular space by many cell types, such as macrophages and activated platelets [11]. It is well-documented that extracellular CyPA is currently involved in the expression of adhesion molecules and induction of the inflammatory responses, including the chemotaxis of monocytes, neutrophil granulocytes, T lymphocytes, and eosinophil granulocytes [12,13]. Importantly, CyPA is recognized to be involved with the formation of several organ fibrosis. In CVB3-induced myocarditis, knockout of CyPA significantly inhibited the infiltration of macrophages and T lymphocytes in the myocardium, whereas inhibition of CyPA by its specific inhibitor—NIM811, could remarkably ameliorate the myocardial fibrosis by modulating its remodeling [14]. Similarly, the NIM811 was also found to be strongly correlated with the reduction of TGF-β1 in liver fibrosis induced by carbon tetrachloride [15]; extracellular CyPA was also observed to activate the SMAD pathway and serve as SMAD-controlled fibrosis mediators in biliary atresia [16]. In this sense, it is strongly believed that CyPA might play a crucial role during the fibrosis pathogenesis in different organs, whereas the mechanism remains unclear, especially in kidney fibrosis.

Extracellular CyPA exerts inflammatory effects through binding to the Extracellular Matrix Metalloproteinase Inducer (EMMPRIN, CD147) in a cell-type specific manner [17]. Sufficient evidence has revealed that the interaction of CyPA and CD147 could trigger a wide array of diseases and pathological processes, including tumor invasion, inflammation, tissue remodeling, and neural dysfunction, by inducing the expression of matrix metalloproteinase 9 (MMP-9) and activating the NF-κB pathway [18–20]. The *Bsg* (mice CD147 gene) promotes the transition of fibroblasts to myofibroblasts by increasing the expression of α-SMA in corneal fibroblasts [21]. Also, a recent study demonstrated that both CyPA and CD147 significantly contribute to renal inflammation, acute kidney injury, and renal cell carcinoma [22]. Furthermore, knockout of *Bsg* is significantly related to the reduced production of MMPs and induction of TGF-β1, thus decreasing renal fibrosis formation [23]. Therefore, interactions between CyPA and CD147 could be strongly suggested to be an essential regulator of fibrosis, including renal fibrosis. However, the effect and mechanism of CyPA/CD147 in renal allograft fibrosis is unknown, which may be considered as a promising target.

In this study, we hypothesized that CyPA/CD147 could promote the progression of renal allograft fibrosis by targeting EMT, which has been identified as a crucial origin of renal fibrosis consequently contributing to the CAD pathogenesis.

## Materials and methods

### Animals and experimental design

Adult male Lewis and F344 rats (200–250 g) were purchased from Charles River Laboratories (Beijing, China) and raised in the Animal Feeding Center of Nanjing Medical University with clean tap water and standard rat chow.

The orthotopic renal transplant surgery was conducted between the donor of Lewis rats and the recipient of F344 rats in the Allo (Allogenic) group (*n* = 5), whereas the Syn (Syngeneic) group was established by transplanting the donor kidney of Lewis rats to the recipient of Lewis rats (*n* = 5). The mean warm and cold ischemia times for the corresponding surgeries were 34.1 and 15.9 min, respectively. Nephrectomy surgeries of the right kidney in the recipient rats were performed after three days post-transplant. Cyclosporine A (5 mg/kg body weight) (Novartis, Switzerland) was administrated once a day intraperitoneally for 14 days post-transplant. The sham surgery was performed on the F344 rats from the same batch, which was considered as a sham group. The recipients were euthanased by head-breaking after 20 post-transplant weeks, and renal allograft tissues were harvested. Then, samples were divided into two parts, which were conserved in liquid nitrogen and fixed in the paraffin, respectively. In addition, the blood samples of the recipient rats in both groups were collected after 4, 12, and 20 post-transplant weeks and stored in liquid nitrogen. Concentrations of serum creatinine and blood urea nitrogen (BUN) were assessed according to the manufacturer’s instructions (Jiancheng, Beijing, China).

### Histology

The collected rat renal allograft tissues from the Allo group (*n* = 5) and the Syn group (*n* = 5) were fixed in 4% paraformaldehyde, embedded in paraffin, and then cut into 2-μm slides. These slides were used for the hematoxylin and eosin (H&E), Masson’s trichome, and immunohistochemistry (IHC) staining as described previously [24]. Briefly, 2-μm sections were stained with H&E during the H&E staining. The morphological changes were observed and evaluated by IF/TA, and the fibrotic area was calculated from five fields of view in each section. The extent of IF/TA, including three categories ranging from I (mild IF/TA) to III (severe IF/TA), was quantitatively evaluated following Banff 2017 criteria in H&E and Masson’s trichome staining [25]. Furthermore, sections were de-paraffinized and rehydrated in a series of alcohol with graded concentrations. The primary antibodies, including rabbit polyclonal anti-E-cadherin (1:100; Abcam, USA), rabbit polyclonal anti-α-SMA (1:200; Abcam, USA), rabbit polyclonal anti-CD147 (1:200; Abcam, USA), and rabbit polyclonal anti-CyPA (1:200; Abcam, USA), were used to incubate with sections overnight at 4 °C. Slices were incubated with goat anti-rabbit IgG (5.0 μg/ml, Abcam, USA) for 1 h. Images from immunohistochemically stained slides were captured. Analysis of positive staining was performed under a light microscope equipped with digital camera (Nikon, ECLIPSE 80i, Japan) by two independent authors (K.L. and X.L.).

### Cell culture and treatment

HK-2 cells, a well-recognized renal tubular epithelial cell line, was purchased from the KeyGen Biotech Company (Nanjing, China) and cultured in the Dulbecco’s modified Eagle’s medium/F12 medium containing 1% penicillin–streptomycin and 10% fetal bovine serum (FBS) in a 5% CO_2_ atmosphere at 37 °C. For the CyPA intervention, starvation of HK-2 cells was performed without any serum overnight and stimulated with two concentrations of CyPA (100 μg/mL, 200 μg/mL) for 5 days, whereas the control group was treated with the solution of CyPA. SiRNA-CD147 plasmid transfection of HK-2 cells was carried out following the manufacturer’s instructions (Invitrogen). The cells were transfected with 1.0 μg of the CD147 plasmid using Lipofectamine 2000 (Invitrogen, USA), and the empty pcDNA3.1 vector was used as a negative control. The MAPK-specific inhibitor, SB203580 (10 μM), was applied ahead to HK-2 cells for 30 min before the intervention of CyPA and/or mAb. In order to explore the mechanism of MAPK signaling pathway in HK-2 cells, four groups were established: (1) cells treated with CyPA and SB203580; (2) cells treated with CyPA and without SB203580; (3) cells treated with SB203580 and without CyPA; and (4) cells treated without CyPA or SB203580. For the detection of p38MAPK phosphorylation, cells in four groups were harvested at 30 min after the intervention, while the expression of α-SMA was examined at 24 h following the intervention. To explore the distribution and expression of α-SMA in HK-2 cells treated with CyPA, cell fluorescence staining was performed. Briefly, cells treated with CyPA 200 μg/mL for 5 days were collected, and primary antibody of α-SMA (1: 200; Abcam, USA) was added into cells for overnight.

### Real-time polymerase chain reaction

Rat kidneys were snap-frozen in liquid nitrogen for total mRNA isolation, whereas total mRNA of HK-2 cells from each group was also extracted using Trizol (Invitrogen, Carlsbad, CA, USA) and the RNA extraction kits (TIANGEN, Beijing, China) according to the manufacturer’s instructions. Briefly, a PrimeScriptTMRT reagent kit (TaKaRa Biotechnology, Japan) was used to synthesize the cDNA. cDNA derived from 0.5 mg of total RNA was amplified by real-time polymerase chain reaction (RT-PCR). The quantitative real-time PCR (qRT-PCR) was conducted by a SYBR Green PCR kit (TaKaRa Biotechnology) under a DNA Engine Opticon 2 System (BioRad Laboratories, Hercules, CA, USA). The primers used for the mRNA evaluation are shown as follows: CD147, 5′-GGAATTCGCCACCATGGCGGCTGCGCTGTTCG-3′ (F) and 5′-CGGGATCCTCAGGAAGAGTTCCTCTGG-3′ (R); CyPA, 5′-CAAGGTCCCAAAGACAGCAGA-3′ (F), 5′-AAG ATG CCA GGA CCC GTA TGC-3′ (R); E-cadherin, 5′-CGAGAGCTACACGTTCACGG-3′ (F), 5′-GGGTGTCGAGGGAAAAATAGG3′ (R); fibronectin (FN), 5′‐ CGGTGGCTGTCAGTCAAAG‐3′(F), 5′‐AAACCTCGGCTTCCTCCATAA‐3′(R); and actin: 5′‐TGACGTGGACATCCGCAAAG‐3′ (F), 5′‐CTGGAAGGTGGACAGCGAGG‐3′ (R). Original data were analyzed using SDS 2.1 software (Applied Biosystems), and fold expression changes and standard errors were calculated using the equation 2-DDCt (Ct, threshold cycle). All the experiments mentioned were carried out at least three times.

### Western blotting

The Western Blot assay was performed as previously reported [24]. Briefly, total proteins were extracted from the rat kidney tissues and HK-2 cells from each group. Samples were separated by 10% SDS-PAGE and then transferred into a nitrocellulose membrane (Whatman, Florham Park, NJ). The membrane was blocked using 5% dry fat-free milk in PBS with 0.1% Tween for 60 min. at room temperature, and then incubated with primary antibodies overnight which are shown as follows: anti-α-SMA antibody (1:1000; Abcam, USA); anti-E-cadherin antibody (1:1000; Abcam, USA); anti-FN antibody (1:1000; Abcam, USA); anti-CD147 antibody (1:1000; Abcam, USA); anti-CyPA antibody (1:1000; Abcam, USA); anti-GAPDH antibody (1:1000; Abcam, USA); anti-phospho-p38 MAPK antibody (1:1000; Cell Signaling Technology, USA); and anti-p38 MAPK antibody (1:1000; Cell Signaling Technology, USA). The secondary antibody used was a goat anti-rabbit IgG (1:4000; Abcam, USA). The proteins were visualized by chemiluminescence reagents (Amersham Biosciences, USA). All the experiments were carried out at least three times.

### Statistical analysis

Data were shown as mean ± standard deviations (SD). Statistical analyses were conducted with the unpaired, two-tailed Student’s *t*-test for single comparisons between two groups; while analysis of variance was performed when compared among more than two groups. A *p*-value < 0.05 was considered statistical significant. All statistical analyses were performed using the SPSS v18.0 system (SPSS Inc., Chicago, IL, USA).

## Results

### CyPA and CD147 are highly expressed in CAD

**Figure 1.**
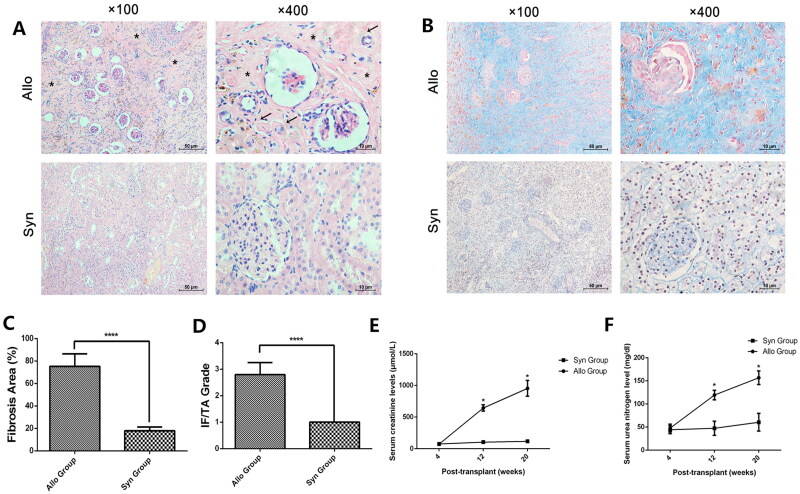
Establishment and identification of rat renal transplant model. We established a 20-week rat renal transplant model with significant chronic allograft fibrosis. The renal allografts from recipient rats were harvested and stored for further examination. H&E staining (A) and Masson staining (B) were performed to identify the interstitial fibrosis (marked by an asterisk in (A)) and tubular atrophy (pointed by arrowhead in (A)) (IF/TA) in the renal allograft. The quantitative analysis of fibrosis area (C) and IF/TA grade (D) was presented. Furthermore, renal allograft function was tested by serum creatinine (E) and blood urea nitrogen (F).

We have established a 20-weeks rat renal transplant model with CAD, and no rat recipient in Allo group and Syn group died during the 20 weeks follow-up. Our results revealed that a remarkable IF/TA, which was considered the crucial pathological characteristics for CAD, was observed in the H&E and Masson’s staining from allograft in the Allo group (Figure 1(A,B)). Moreover, significant infiltration of mononuclear cells among the IF/TA was also found. The quantitative analysis of fibrosis area and IF/TA grade between two groups supported identifying the rat renal transplant CAD model (Figure 1(C,D)). Consequently, we analyzed the allograft function by serum creatinine and BUN, and the results revealed that renal allograft function was significantly reduced in a time-dependent manner in the Allo group compared with the sham subjects (Figure 1(E,F)).

**Figure 2.**
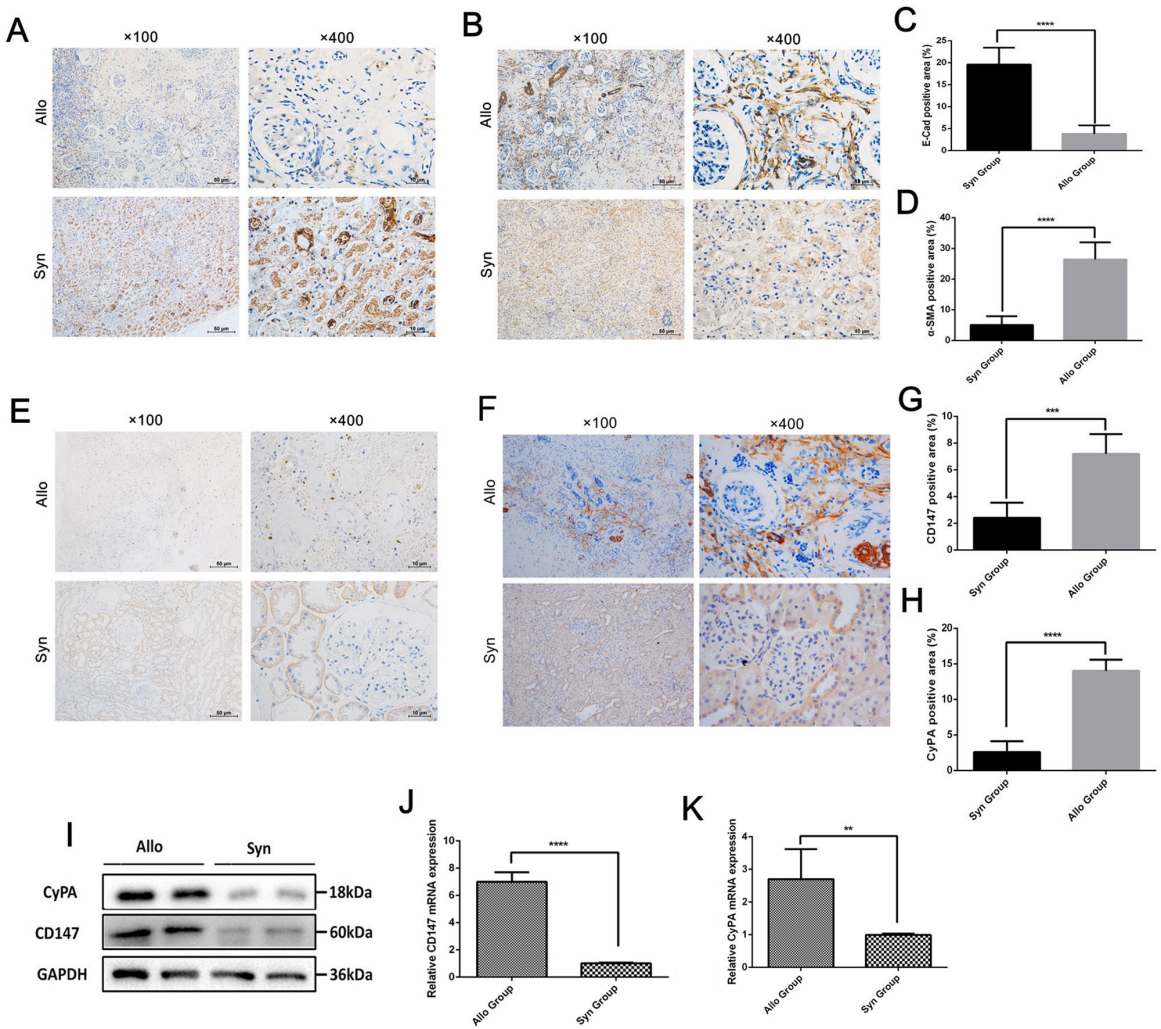
The expression of CyPA and CD147 in rat renal transplant model. We collected the renal allograft tissue and performed the immunohistochemistry staining of E-cadherin (A) and α-SMA (B) to examine the occurrence of epithelial-to-mesenchymal transition in chronic allograft dysfunction, as well as the expression of CD147 (C) and CyPA (D) in renal allograft tissue. In addition, the protein and mRNA of CD147 and CyPA were also tested (E and F).

We tested the protein expression of E-cadherin and α-SMA, the biomarkers of EMT, in the allograft tissues. The results showed that the α-SMA expression was upregulated, and the E-cadherin expression was decreased, suggesting the existing presence of EMT progression in CAD (Figure 2(A,B)). The quantitative results of E-cadherin and α-SMA between two groups are presented in Figure 2(C,D). Then, we designed an experiment to explore the protein distribution and expression of CyPA and CD147 in the pathogenesis of CAD. Enhanced expression of CyPA and CD147 were identified in 20-weeks renal allograft tissues, mainly distributed in the allograft tubular epithelial regions (Figure 2(E,F)), the quantitative results of which are shown in Figure 2(G,H). Furthermore, we examined the total protein and the mRNAs expression of CyPA and CD147 in allograft tissue. The results were consistent with those in IHC staining (Figure 2(I–K)).

### CyPA/CD147 signaling induces the progression of EMT

**Figure 3.**
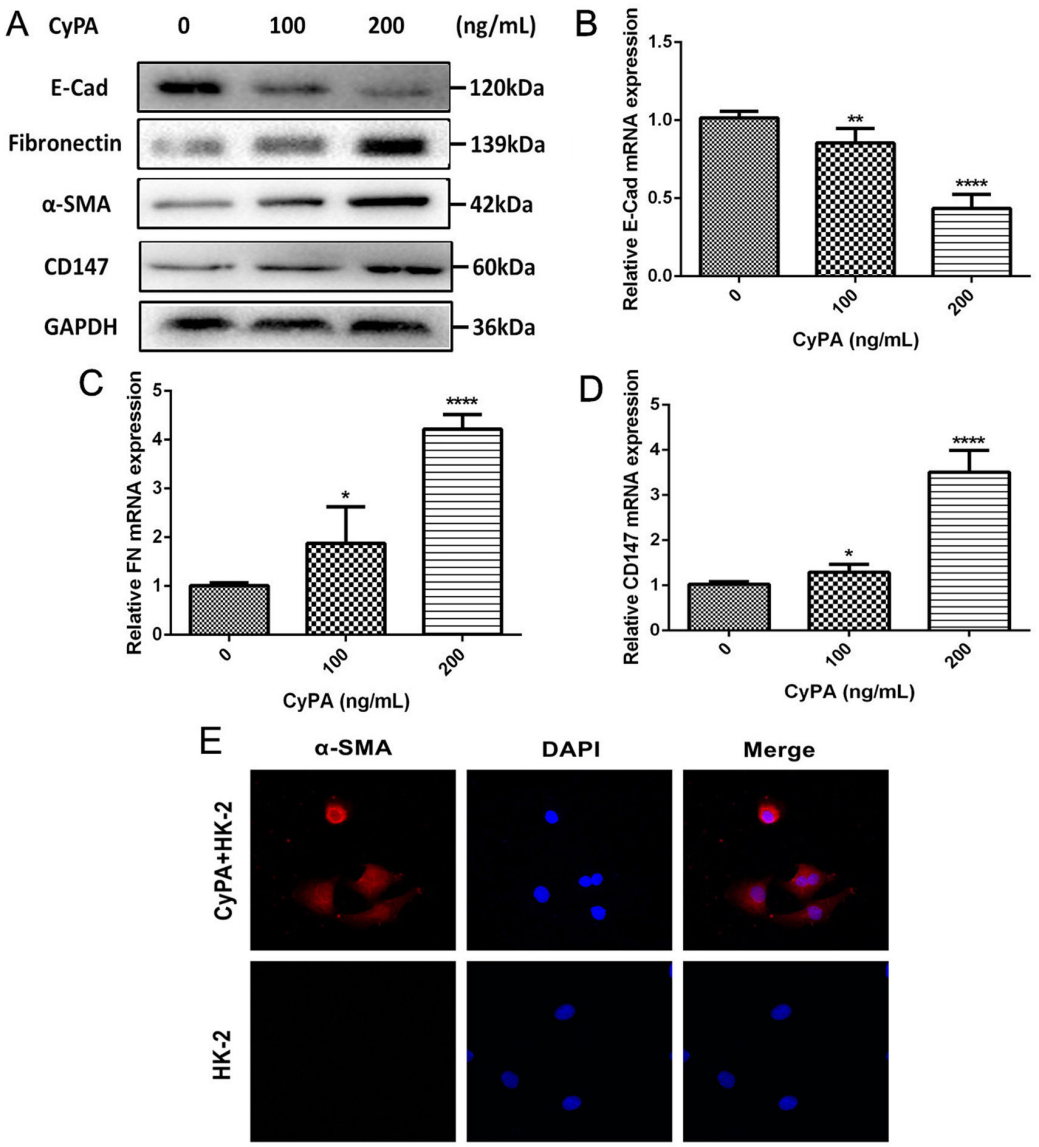
The effect of CyPA on CD147 and tubular fibrosis in HK-2 cells. We stimulated the HK-2 cells by various concentrations of CyPA and extracted the total protein and mRNA from cells. The CD147 protein, as well as the biomarkers related to tubular fibrosis, were tested by Western Blot assay (A). Moreover, the mRNA expression of E-cadherin (B), fibronectin (FN; C), and CD147 (D) were explored by RT-PCR. The cell fluorescence staining of α-SMA was presented (E).

CD147 is mainly present in renal tubular cells, evidenced by our results in histological examinations [26]. To clarify the CyPA/CD147 role during the EMT and renal fibrosis progression, we performed an *in vitro* study. Western blotting results revealed that the protein expressions of CD147, α-SMA, and FN were remarkably higher after the stimulation of CyPA in a dose-dependent manner in HK-2 cells, whereas the E-cadherin expression was conversely reduced (Figure 3(A,B)). Similar results were found in the mRNA expression (Figure 3(C,D)). In the cell fluorescence staining, the α-SMA was observed to be highly expressed in HK-2 cells treated with CyPA when compared with the control group (Figure 3(E)).

**Figure 4.**
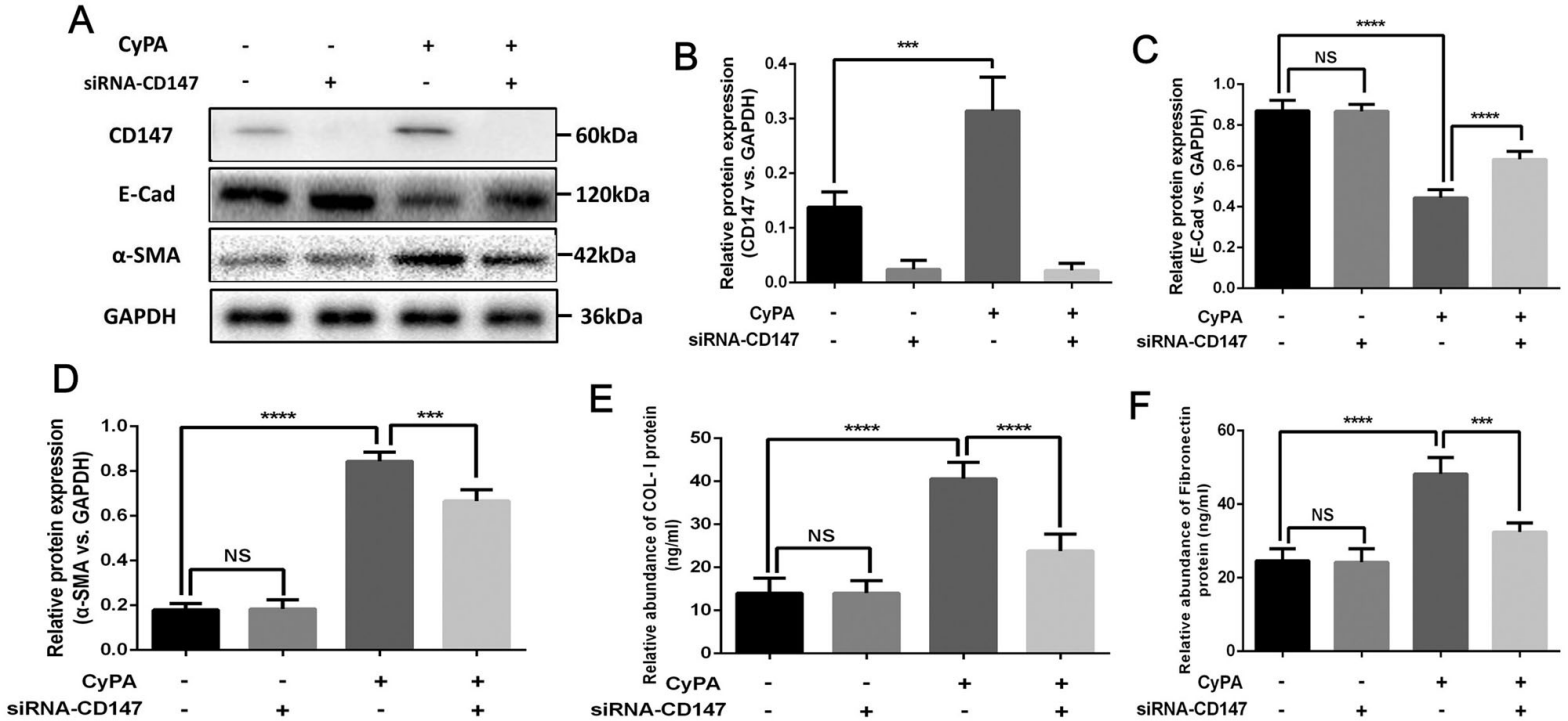
The identification of the effect of CyPA on the CD147 expression in HK-2 cells. We conducted the siRNA-CD147 and stimulated the HK-2 cells with or without CyPA to block the expression of CD147 (A). The relative expression of CD147 (B), E-cadherin (C), and α-SMA (D) were shown.

Next, we blocked the interactions between CyPA and CD147 to further confirm the effect of CyPA on EMT and renal fibrosis. The siRNA-CD147 intervention significantly blocked the CD147 expression in HK-2 cells, whereas the positive regulation of α-SMA and negative regulation of FN were also reversed (Figure 4(A–D)). In addition, the relative abundance of Collagen-I and FN in the supernatant in each group were examined and the results were consistent with protein test (Figure 4(E,F)), suggesting that the progression of EMT could be induced by CyPA/CD147 signaling.

### CyPA/CD147/p38 MAPK signaling pathway contributes to the progression of EMT and renal fibrosis

**Figure 5.**
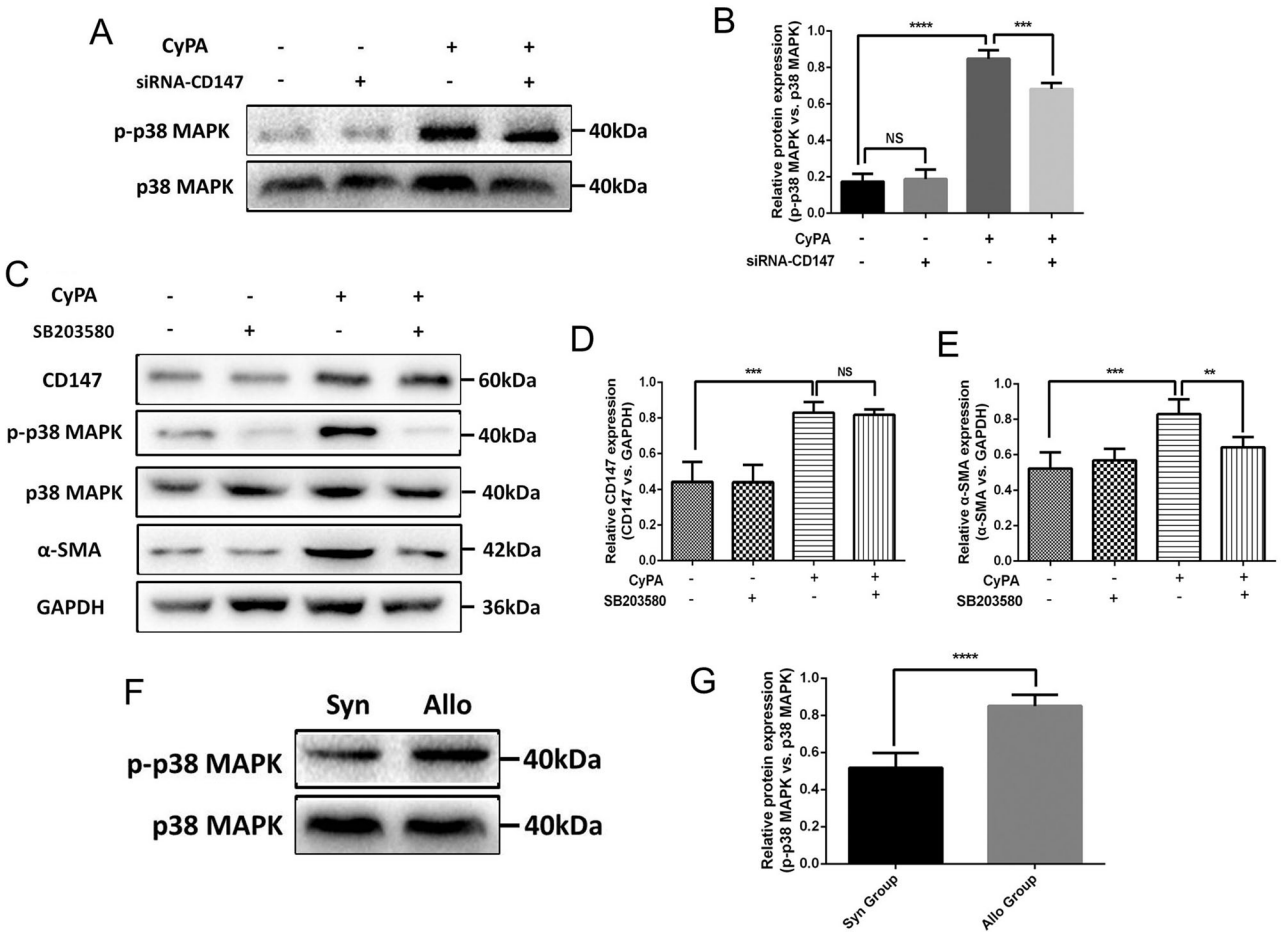
The study of the signaling pathway involved in the CyPA/CD147 interactions in HK-2 cells. We used the siRNA-CD147 to explore the p38 MAPK signaling pathway during the CyPA/CD147 interactions in HK-2 cells (A). Then, we selectively inhibited the expression of p38 MAPK by SB203580 and examined the expression of CD147 and α-SMA (B). The relative expression of CD147 and α-SMA were quantitatively shown (C and D).

It is reported that p38 MAPK signaling pathway participates in the regulation of CyPA and CD147 [27]. Hence, we further explored the potential mechanism involved in the effect of CyPA/CD147 signaling on EMT and renal fibrosis progression. First, we tested the activation of the MAPK signaling in the CyPA/CD147 interactions. Figure 5(A) shows a significant activation of the phosphorylated p38 MAPK, which was the critical mediator of MAPK signaling. Then, we used the specific inhibitor for MAPK signaling, SB203580, to confirm the activation of the MAPK signaling. We observed that upon the intervention of SB203580, the expression of CD147 showed no significance (Figure 5(C,D)), whereas the expression of α-SMA was remarkably inhibited (Figure 5(C,E)), suggesting the downstream role of p38MAPK signaling among the interaction of CyPA/CD147. To further identify the MAPK signaling in the renal fibrosis, the phosphorylation of p38 MAPK in rat renal transplant model was tested, and the significant activation of phosphorylated p38 MAPK was observed (Figure 5(F,G)).

## Discussion

Our *in vivo* and *in vitro* studies revealed that CyPA/CD147 signaling were significantly activated in renal allograft fibrosis and CAD progression. Moreover, the CyPA/CD147 could remarkably induce the process of EMT by p38 MAPK signaling and then contribute to the renal allograft fibrosis, indicating that the CyPA/CD147 could serve to be an essential target for renal allograft fibrosis. To the best of our knowledge, this is the first study to investigate the role of CyPA/CD147 signaling in renal allograft fibrosis.

Several reports investigated the cellular mechanisms of CAD in renal transplant, factors that could be divided into immunological and non-immunological factors [28]. Among these, EMT was identified to play a primary role in the pathogenesis of renal allograft fibrosis by inducing the expression of α-SMA and extracellular matrix, including collagen-I and FN [7]. Notably, the EMT inducers during the formation of CAD were reported as TGF-β1, TNF-α, and renal interstitial inflammation [2,3]. In this study, CyPA could be considered a novel inducer, stimulating the renal allograft fibrosis by interacting with CD147 on the renal tubular epithelial cells, suggesting it as a promising target in the treatment of CAD. Moreover, we have identified that EMT induced by CyPA/CD147 contributes to renal allograft fibrosis, which was consistent with the specific role of EMT in renal fibrosis. Also, blockade of CD147 could attenuate the progression of EMT and renal fibrosis, suggesting the downstream effect of EMT in the regulation of CD147. Similarly, a study focusing on renal fibrosis in the unilateral ureteral obstruction (UUO) mice model provides convincing outcomes of Bsg/CD147 in promoting the development of renal fibrosis by knocking out the *Bsg* in mice, which was consistent with our findings [23]. Compared with this study, we also noticed that EMT could serve as a key downstream process involved in the regulation of CyPA/CD147 in renal fibrosis. Interestingly, we observed a significant infiltration of mononuclear cells and highly expressed CyPA/CD147 distributed in the IF/TA of rat model with CAD. Concerning the effect of CyPA/CD147 in inflammatory responses, we considered a potential link of CyPA/CD147 and immunological cell infiltration during the CAD process, which remained to need further analysis. Therefore, our findings supported the CD147 as a candidate target molecule for the treatment of renal fibrosis, especially in renal allograft fibrosis.

We have identified that CyPA might have a direct pro-fibrotic effect on the tubular epithelial cells. The canonical stimulation for renal fibrosis has been recognized as TGF-β1, TNF-α, MCP-1, and CXCL-10 [29,30]. Considering the critical role of CD147 in regulating renal fibrosis, the CyPA binding to the CD147 was also noticed to be involved in the stimulation of renal fibrosis. Moreover, extracellular CyPA has been described to induce the chemotaxis of T cells, monocytes, and macrophages, which leads to the massive recruitment and infiltration of inflammatory cells in the renal allograft tissues [14,18]. It is well known that specific pro-fibrotic cytokines could be produced and secreted during the inflammatory response, triggering the pathogenesis of organ fibrosis. A recent study using the CyPA knockout mice reported that CyPA could promote neutrophil and macrophage accumulation and kidney damage but fail to lead to the renal fibrosis [31]. With regard to the important role of immune-related factors in the development of renal allograft fibrosis, the pro-inflammatory effect of CyPA may provide extensively supportive effects on the pro-fibrotic formation in kidney transplantation. Therefore, the indirect pro-fibrotic effect of CyPA should also draw great attention even though the crucial role of CyPA/CD147 interaction in renal fibrosis.

A recent study has reported the positive relation of CyPA/CD147 signaling and osteoclast-related MMP-9 expression in mice inflammatory periapical lesions progression [18]. Additionally, CyPA inhibitor NIM811 significantly reduced MMP-9 secretion during the differentiation process of foam cells, suggesting the potential association of the CyPA/CD147 and MMP-9 expression. Significantly, suppressed activities of MMP-2 and MMP-9 proteins in the *Bsg*–/– UUO mice were observed, providing direct evidence for the CyPA in the regulation of MMP-9 expression [23]. Thus, we believed that except for the EMT process, the MMP-9-related signaling induced by CyPA/CD147 interactions also plays a vital role in the extracellular matrix deposition and degradation, and consequently renal fibrosis. In the *in vitro* study, we used the siRNA-CD147 to block the interactions between CyPA and CD147 selectively and observed the attenuation of EMT and renal fibrosis, which was consistent with other studies and our *in vivo* study. In contrast, Sarró et al. [32] demonstrated the different effects of CypA and CypB on the tubular epithelial cells treated with TGF-β1, which seems to be inconsistent with our *in vitro* results. In our study, we designed to explore the direct effect of CyPA on the EMT and renal fibrosis, instead of the treatment of TGF-β1. Unfortunately, we could not perform the intervention of siRNA-CD147 in the rat CAD model or the *Bsg*–/– CAD model to explore and confirm the unique effect of CD147 in the renal allograft fibrosis and CAD.

Other factors also limited our findings. First, only p38 MAPK signaling was included for the cellular mechanism research, which should be comprehensively explored by more extensive tools, such as second-generation sequencing and transcriptomics. Then, we have studied the CyPA/CD147 interactions in the rat renal transplant model, lacking the human samples to be included. Finally, the mechanisms involved between the CD147 and MAPK signaling remain unclear, which should be further explored.

## Conclusions

Our study reported the significantly expressed CyPA/CD147 in the renal allograft fibrosis tissues and suggested the potential association of CyPA/CD147 signaling with CAD. Moreover, the *in vitro* study revealed that CyPA could remarkably promote the EMT process by interactions with CD147 and triggering the intracellular p38 MAPK signaling pathway, contributing to renal fibrosis. Our data provide evidence that CyPA is critically involved in the pathophysiology of renal allograft fibrosis, and CyPA/CD147 signaling might represent a target to modulate the renal allograft fibrosis and CAD.

## Data Availability

The data that support the findings of this study are available from the corresponding author upon reasonable request.
